# The Elk1/MMP-9 axis regulates E-cadherin and occludin in ventilator-induced lung injury

**DOI:** 10.1186/s12931-021-01829-2

**Published:** 2021-08-23

**Authors:** Zhao Tao, Yan Jie, Zhang Mingru, Gu Changping, Yang Fan, Wu Haifeng, Wang Yuelan

**Affiliations:** 1 Department of Anesthesiology and Perioperative Medicine, Shandong Qianfoshan Hospital, Cheeloo College of Medicine, Shandong University, No. 16766 Jingshi Road, Jinan, 250014 Shandong China; 2 Department of Anesthesiology, People’s Hospital of Rizhao, Jining Medical University, No. 126 Tai’an Road, Rizhao, 276826 Shandong China; 3 Department of Anesthesiology and Perioperative Medicine, The First Affiliated Hospital of Shandong First Medical University, Shandong Institute of Anesthesia and Respiratory Critical Care Medicine, No. 16766 Jingshi Road, Jinan, 250014 Shandong China

**Keywords:** Ventilator-induced lung injury, Elk1, MMP-9, Tight junctions

## Abstract

**Background:**

Ventilator-induced lung injury (VILI) is a common complication in the treatment of respiratory diseases with high morbidity and mortality. ETS-domain containing protein (Elk1) and Matrix metalloproteinase (MMP) 9 are involved in VILI, but the roles have not been fully elucidated. This study examined the mechanisms of the activation of MMP-9 and Elk1 regulating barrier function in VILI in vitro and in vivo.

**Methods:**

For the in vitro study, Mouse lung epithelial cells (MLE-12) were pre-treated with Elk1 siRNA or MMP-9 siRNA for 48 h prior to cyclic stretch at 20% for 4 h. For the in vivo study, C57BL/6 mice were pre-treated with Elk1 siRNA or MMP-9 siRNA for 72 h prior to 4 h of mechanical ventilation. The expressions of Elk1, MMP-9, Tissue inhibitor of metalloproteinase 1 (TIMP-1), E-cadherin, and occludin were measured by Western blotting. The intracellular distribution of E-cadherin and occludin was shown by immunofluorescence. The degree of pulmonary edema and lung injury were evaluated by Hematoxylin–eosin (HE) staining, lung injury scores, Wet/Dry (W/D) weight ratio, total cell counts, and Evans blue dye.

**Results:**

20% cyclic stretch and high tidal volume increases the expressions of Elk1, MMP-9, and TIMP-1, increases the ratio of MMP-9/TIMP-1, decreases the E-cadherin and occludin level. Elk1 siRNA or MMP-9 siRNA reverses the degradations of E-cadherin, occludin, and the ratio of MMP-9/TIMP-1 caused by cyclic stretch. Elk1 siRNA decreases the MMP-9 level with or not 20% cyclic stretch and high tidal volume.

**Conclusions:**

The results demonstrate mechanical stretch damages the tight junctions and aggravates the permeability in VILI, Elk1 plays an important role in affecting the tight junctions and permeability by regulating the balance of MMP-9 and TIMP-1, thus indicating the therapeutic potential of Elk1 to treat VILI.

## Background

Ventilator-induced lung injury (VILI) is mainly caused by the destruction of lung tissue, interstitial structures and alveolar membrane injury due to excessive alveolar dilatation or excessive intrapulmonary pressure [1–3]. VILI is primarily found in patients submitted to general anesthesia and/or intensive care units. The pathogenesis of VILI is based on increased permeability of the alveolar capillary membrane, which is associated with reduced or inactivated pulmonary surfactant and increased alveolar permeability and inflammation [4–6]. The clinical features of VILI include decreased lung compliance, pulmonary edema and impaired oxygenation, thus lead to acute respiratory distress syndrome [7, 8]. Therefore, more research is needed to better understand how to protect against lung injury caused by mechanical ventilation.

In this regard, tight junctions, which connect adjacent cells through tight protein particles, play an important role in alveolar permeability [9–11]. Our previous studies have found that high tidal volume mechanical ventilation can decrease the expressions of tight junctions E-cadherin and occludin, thus increasing alveolar permeability [12, 13]. The pathways regulating tight junctions have not been fully investigated. Therefore, we aimed to identify the activation mechanism of E-cadherin and occludin in VILI.

Matrix metalloproteinase 9 (MMP-9) is thought to regulate the dynamic balance of extracellular matrix, neutrophil transmigration and barrier function [14–16]. TIMP1 is an MMP9-specific tissue inhibitor, the proportion of MMP-9/TIMP-1 in the lung and synthesis and degradation of the cell junctions is in a state of dynamic equilibrium [17]. Upregulation the ratio of MMP-9 and TIMP-1 damaged the alveolar–capillary membrane and pulmonary edema in lipopolysaccharide-induced acute lung injury [18]. In VILI, increased MMP-9 in neutrophil transmigration was found to cause lung injury [19, 20]. However, it is unclear that the balance of MMP-9 and TIMP-1 regulated occludin and E-cadherin in VILI. Therefore, we aimed to identify that.

ETS-domain containing protein (Elk1) is a transcription factor involved in regulating the expression of various genes involved in cellular growth, proliferation, apoptosis, tissue remodeling and angiogenesis [21]. Elk1 enhances its binding activity with DNA by its ETS domain, thus connecting the target gene promoter to regulate the expression and activity of downstream proteins [22]. Interestingly, the MMP-9 promoter region has ETS binding sites [23]. Therefore, we aimed to assess a possible association between Elk1 and MMP-9 in VILI.

Collectively, we hypothesized that high tidal volume mechanical ventilation could activate Elk1, thus upregulating the ratio of MMP-9/TIMP-1, which would inhibit E-cadherin and occludin in VILI.

## Methods

### Cell culture and treatment

Mouse lung epithelial cells (MLE-12) were purchased from American Type Culture Collection (Manassas, VA, USA). MLE-12 cells (a density of 5 × 10^5^ cells/mL) were seeded on collagen I-coated flexible-bottom BioFlex plates with 10% fetal bovine serum, penicillin (100 U/ml), and streptomycin (100 mg/ml) at 37 °C in a humidified atmosphere containing 5% CO_2_. MLE-12 cells were randomly divided into the following groups: CS(0 h) group, CS(2 h) group, CS(4 h) group, si-Nc group, si-Elk1 group, si-MMP-9 group, CS(4 h) + si-Nc group, CS(4 h) + si-Elk1 group, and CS(4 h) + si-MMP-9 group.

Elk1 siRNA (si-Elk1), MMP-9 siRNA (si-MMP-9) and Negative control siRNA (si-Nc) were constructed and synthesized by Gene Pharma Corporation. Si-Nc was transfected into 50% confluent MLE-12 cells with the Lipofectamine 3000 transfection reagent in si-Nc group and CS(4 h) + si-Nc group, as si-Elk1 transfected in si-Elk1 group and CS(4 h) + si-Elk1 group, si-MMP-9 transfected in si-MMP-9 group and CS(4 h) + si-MMP-9 group. After transfection for 48 h, MLE-12 cells were treated with cyclic stretch in CS(4 h) + si-Nc group, CS(4 h) + si-Elk1 group, and CS(4 h) + si-MMP-9 group. For the cyclic stretch, we used the FX-5000 T Flexercell Tension Plus system, and its parameters (VILI model in vitro) were set as follows: 20% stretch amplitude, a frequency of 30 cycles/min, a stretch-to-relaxation ratio of 1:1 applied in a cyclic manner, time of cyclic stretch for 0, 2, or 4 h. CS(0 h) group, CS(4 h) group, or si-Nc group was as the control group in different studies. Three experiments were conducted at least.

### Animals

Fifty male C57BL/6 mice that weighed 20–25 g (6–8 weeks of age) were purchased from Vital River Laboratory and housed under specific pathogen-free conditions. All animal procedures were performed in accordance with the established guidelines, reviewed and approved by the Laboratory Animal Ethics Committee of Qianfoshan Hospital of Shandong University. Mice were randomly divided into five groups (10 mice per group): MV(0 h) group, MV(2 h) group, MV(4 h) group, MV(4 h) + si-Elk1 group, and MV(4 h) + si-MMP-9 group. All animals were treated with trachea cannula. The animals in the MV(0 h) group were treated with trachea cannula only, the mice in other groups were exposed to mechanical ventilation for 2 or 4 h. MV(0 h) group was the control group. Including criteria were male, grade of specific pathogen-free, weight of 20–25 g, and age of 6–8 weeks. Excluding criteria were death in the process of transfection, trachea cannula, or mechanical ventilation. Five molecular biology experiments were conducted at least.

### VILI model in vivo and experimental protocol

All mice received the same standard diet during the experimental period. The mice were anesthetized via an intraperitoneal injection of pentobarbital and ketamine. Pancuronium was used to maintain muscle relaxation. The ventilation parameters (VILI model in vivo) were set as follows: tidal volumes of 20 mL/kg, a respiratory rate of 40 times/min, PEEP of 0 cm H_2_O, an inspiratory-to-expiratory (I/E) ratio of 1:2, a fraction of inspired oxygen of 21% and a time of mechanical ventilation of 0, 2, or 4 h.

For si-Elk1 or si-MMP-9 in vivo transfection, mice were injected by caudal vein with si-Elk1 (2.5 μg/g) or si-MMP-9 (2.5 μg/g), 10% glucose solution, DEPC water, and the Entranster™ In Vivo Transfection reagent (Engreen, 18668-11-1, Beijing, China) at the dosage of 200 µL according to the manufacturer’s instructions. Animals were fasted for 24 h before experiments and given free access to water. After 72 h of transfection, the mice were treated with mechanical ventilation for 4 h.

### Bronchoalveolar lavage fluid (BALF)

After anesthesia and mechanical ventilation, the mice were treated with precooled saline (0.3 mL) into the lungs injected by tracheal intubation, and then pumped back after three seconds. The same operation was repeated three times. The number of cells was measured in the BALF.

### Histopathological analysis

After the mice were treated with mechanical ventilation, the lungs were inflated manually at the pressure of 30 cmH_2_O for three times, 10 s each time, and then euthanized. The lung injury scores were recorded. The upper lobe of the right lung was fixed in 4% paraformaldehyde for 72 h and embedded in paraffin. Tissue blocks were cut into 5-μm slices, stained with hematoxylin for 5 min and eosin for 2 min (HE). The HE staining sections were observed under a light microscope at a magnification of 400 × .

### Lung wet/dry (W/D) weight ratio

After mechanical ventilation, the left lung was collected after heparin was injected through the postcava vein. The lung was weighed to determine the wet lung weight, then dried at 65 °C for 48 h and weighed again to determine the dry weight. The lung W/D ratio was calculated.

### Evans blue dye extravasation

The Evans blue dye was injected by the tail vein 1 h before mice were euthanized. The lungs were transferred, weighed, incubated with formamide (500 μL) at 55 °C for 24 h to extract the dye. The formamide/Evans Blue mixture was centrifuged to pellet any remaining tissue fragments. The optical density was determined spectrophotometrically at 620 nm. The results were expressed as nanograms of dye per microgram of wet tissue.

### Immunofluorescences

MLE-12 cells and lung tissue sections were permeabilized with immunostaining permeabilization buffer containing saponin for 5 min, then blocked with 5% BSA for 30 min at room temperature, and incubated with anti-E-cadherin (1:100, Santa Cruz Biotechnology, Dallas, TX, USA) and anti-occludin (1:100, Abcam, Cambridge, MA, USA) antibodies diluted in 5% BSA overnight at 4 °C. The specimens were incubated with green-fluorescent Alexa Fluor 488 donkey anti-rabbit IgG or red-fluorescent Alexa Fluor 594 rabbit anti-mouse IgG (Invitrogen, Grand Island, NY, USA) at room temperature for 1 h after primary antibodies were washed off. The nuclei were stained with 4′,6-diamidino-2-phenyl indole dihydrochloride (DAPI) for 5 min. A high-sensitivity laser confocal microscope was used to observe the changes of E-cadherin and occludin.

### Western blotting and immunoprecipitation

After mechanical ventilation or cyclic stretch, the remaining right lung tissue or MLE-12 cells respectively were lysed on ice in a mixture of RIPA and PMSF (Beyotime, China). Equal amounts of protein were separated by 8% sodium dodecyl sulfate polyacrylamide gel electrophoresis, transferred to a polyvinylidene difluoride membrane, blocked in 5% nonfat milk for 2 h at temperature, and incubated with primary antibodies at 4 °C overnight. The primary antibodies were as follows: E-cadhrein (1:500), occludin (1:1000), Elk1 (1:500, Abcam, Cambridge, MA, USA), MMP-9 (1:500, Santa Cruz Biotechnology, Dallas, TX, USA), TIMP-1 (1:500, Santa Cruz Biotechnology, Dallas, TX, USA), and GAPDH (1:1000, Abcam, Cambridge, MA, USA). The membranes were incubated with goat anti-mouse or goat anti-rabbit secondary antibodies for 2 h at room temperature. The ECL SuperSignal reagent (Millipore, Billerica, CA, USA) was used to detect the protein bands by FluorChem E (ProteinSimple, CA, USA). The AlphaView software (ProteinSimple, CA, USA) was used to analyze the relative densities of the proteins. All experiments were performed in triplicates.

After treatment with cyclic stretch for 0 or 4 h, MLE-12 cells were lysed in buffer with a protease cocktail for 30 min. The lysate was separated by centrifugation for 30 min. A small amount of supernatant was used for input, and the other part was precleared using isotype control IgG and protein A/G plus-agarose beads (20 μL) for 4 h, and then incubated with anti-Elk1 antibody and protein A/G plus-agarose beads (20 μL) at 4 °C overnight. The immunoprecipitated proteins were dissolved in 2 × loading buffer for immunoblot analysis.

### Statistical analysis

All data are expressed as mean ± SD, and obtained from three independent experiments at least. Significant differences were assessed using one-way analysis of variance (ANOVA) and least significant difference test for multiple group comparisons. Statistical analysis was performed using the SPSS 26.0 statistics package for windows. A *P*-value < 0.05 was considered statistically significant.

## Results

### In vivo and in vitro VILI models present the activation of Elk1 and MMP-9 and loss of E-cadherin and occludin

**Fig. 1:**
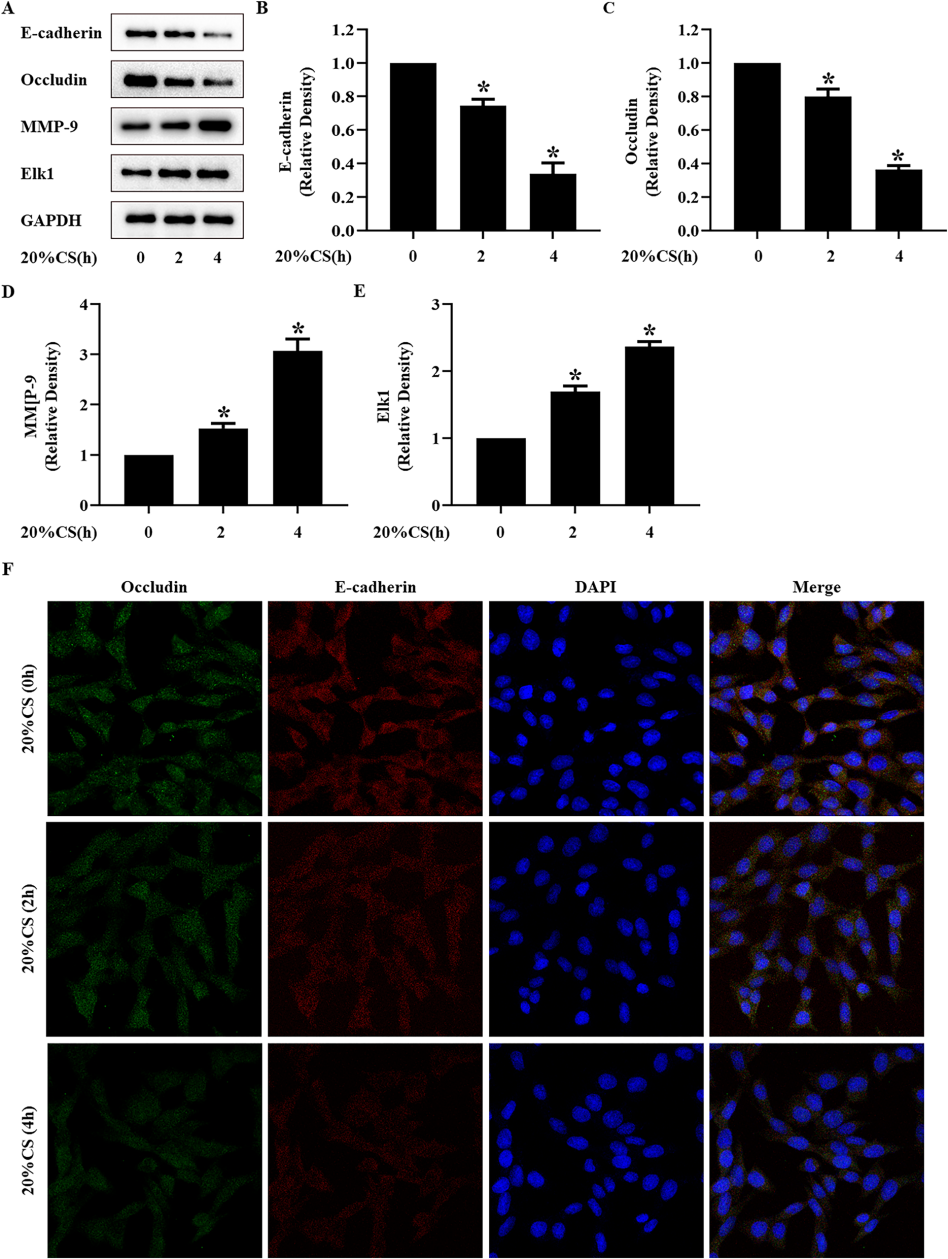
20% cyclic stretch induced Elk1 and MMP-9 activation and occludin and E-cadherin degradation in MLE-12 cells. MLE-12 cells were treated with 20% cyclic stretch for 0, 2, and 4 h. **A** Representative Western blotting of E-cadherin, occludin, Elk1, and MMP-9. **B**–**E** The density of the proteins at 0 h was used as a standard. *P < 0.05, vs. the CS(0 h) group. F: Immunofluorescence was used to detected the distribution of occludin (green) and E-cadherin (red), and the nuclei were stained with DAPI (blue). All experiments were repeated at three times

**Fig. 2:**
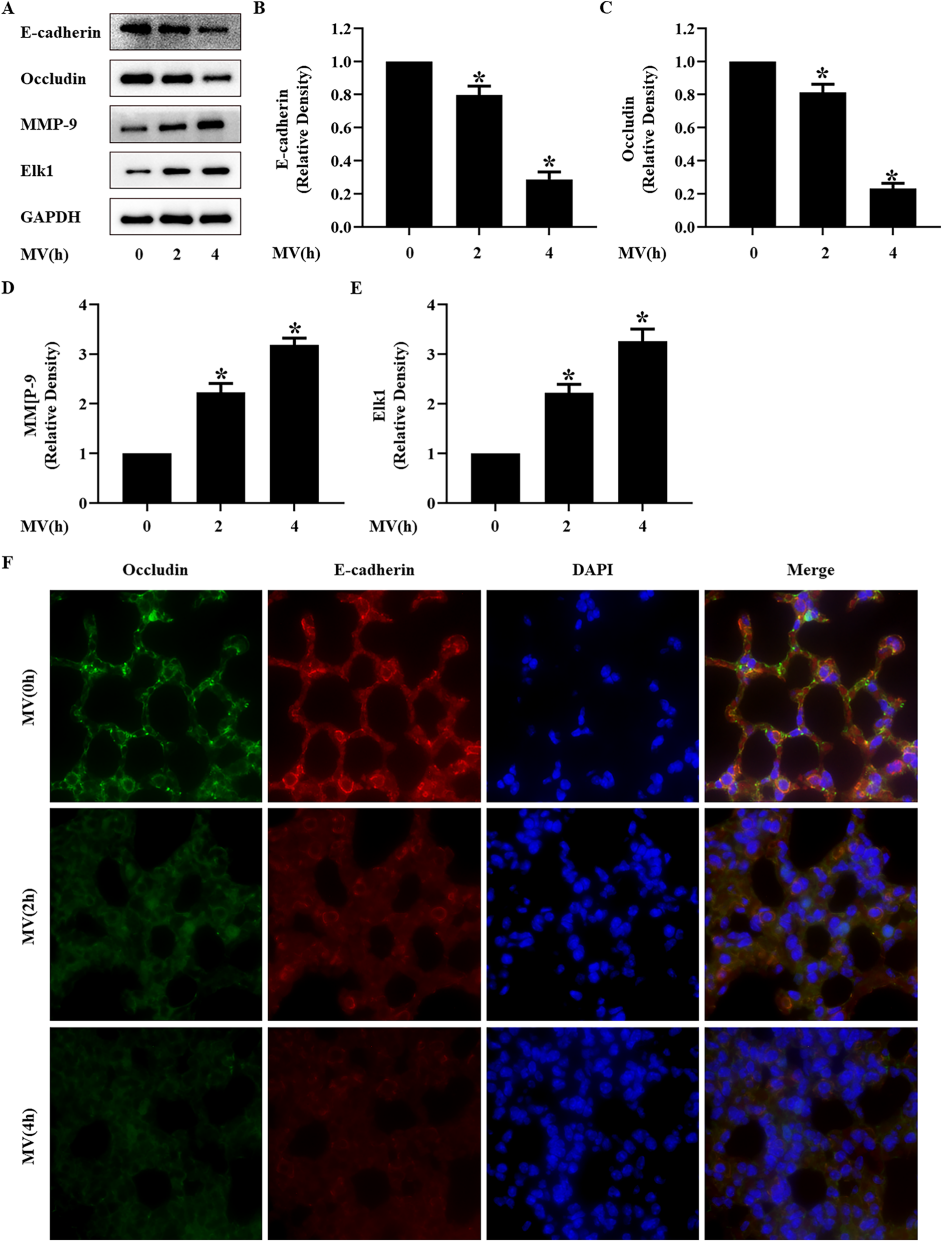
High tidal volume mechanical ventilation induced Elk1 and MMP-9 activation and occludin and E-cadherin degradation in C57BL/6 mice. C57BL/6 mice were treated with high tidal volume (20 mL/kg) for 0, 2, and 4 h. **A** Representative Western blotting of E-cadherin, occludin, Elk1, and MMP-9. **B**–**E** The density of the proteins at 0 h was used as a standard. *P < 0.05, vs. the MV(0 h) group. **F** Immunofluorescence was used to detected the distribution of occludin (green) and E-cadherin (red), and the nuclei were stained with DAPI (blue). All experiments were repeated at five times

In vivo*,* mice were treated with a high tidal volume (20 mL/kg) for 0, 2, and 4 h, while in vitro*,* MLE-12 cells were treated with a 20% cyclic stretch for 0, 2 and 4 h. The expressions of Elk1 and MMP-9 were increased, while E-cadherin and occludin decreased in a time-dependent manner after mechanical ventilation or cyclic stretch (Figs. 1A–E, 2A–E). The immunofluorescence of lungs and MLE-12 cells showed that the intracellular distribution of E-cadherin and occludin decreased in a time-dependent manner (Figs. 1F, 2F).

### Elk1 or MMP-9 knockdown regulated the loss of E-cadherin and occludin in VILI

**Fig. 3:**
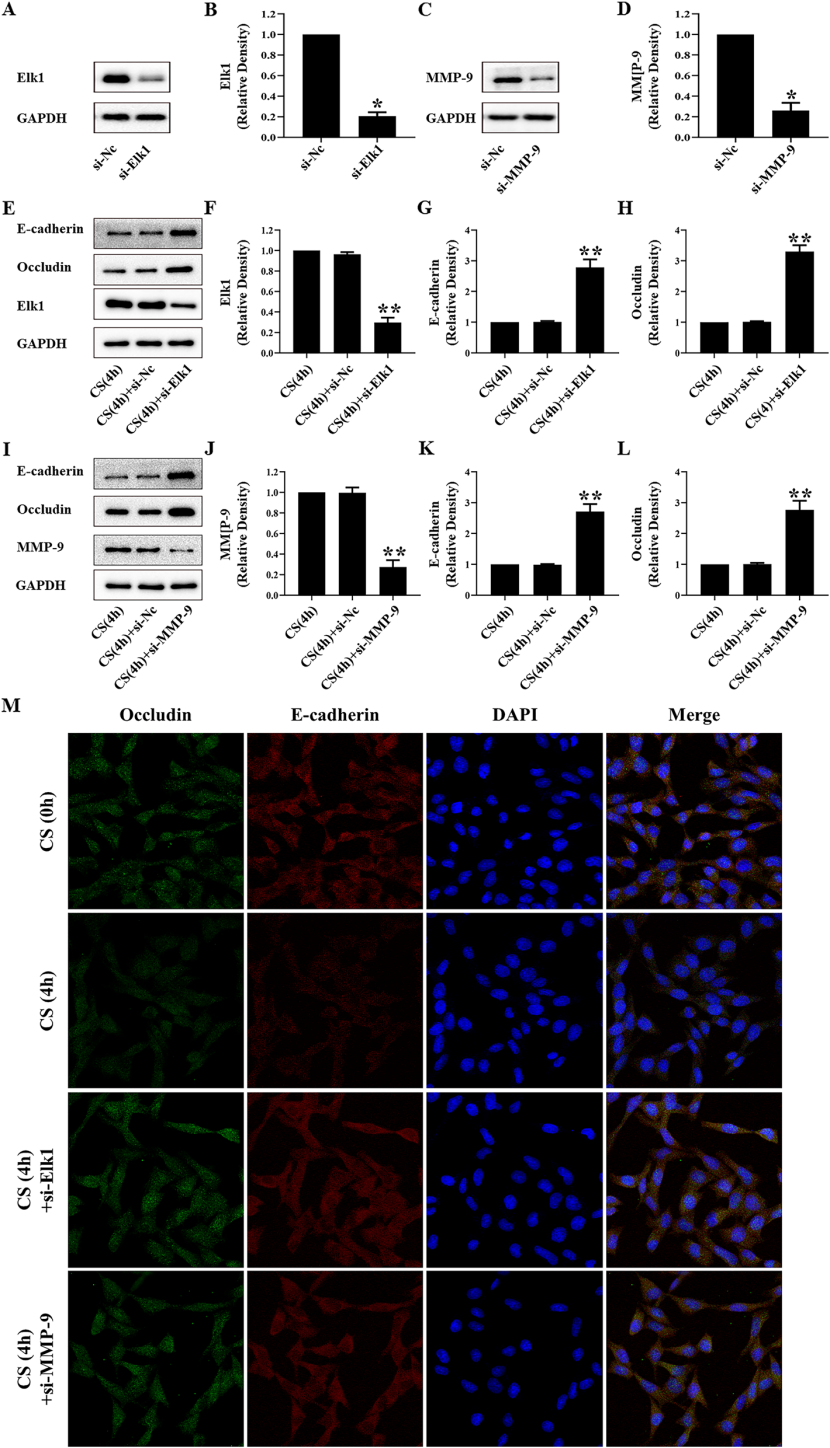
MMP-9 and Elk1 mediated the degradation of occludin and E-cadherin after 20% cyclic stretch. **A** and **C** Representative Western blotting of Elk1 and MMP-9 in MLE-12 cells treated with si-Elk1 or si-MMP-9. **B** and **D** The density of the proteins at si-Nc was used as a standard. *P < 0.05, vs. the si-Nc group. E and I: Representative Western blotting of Elk1, MMP-9, E-cadherin, and occludin in MLE-12 cells treated with si-Elk1 or si-MMP-9 and 20% cyclic stretch for 4 h. **F**–**L** The density of the proteins at the CS(4 h) group was used as a standard. **P < 0.05, vs. the CS(4 h) group. M: Immunofluorescence was used to detected the distribution of occludin (green) and E-cadherin (red), and the nuclei were stained with DAPI (blue) in MLE-12 cells treated with si-Elk1 or si-MMP-9 and 20% cyclic stretch for 4 h. All experiments were repeated at three times

In vitro, MLE-12 cells were pre-treated with si-Elk1 or si-MMP-9 and subjected to a 20% cyclic stretch for 4 h. The expression of Elk1 was 80% lower in si-Elk1 cells, while there was a 70% decrease in si-MMP-9 cells when compared to normal cells (Fig. 3A–D). Both si-Elk1 and si-MMP-9 abolished the down-regulation of E-cadherin and occludin induced by cyclic stretch (Fig. 3E–L). The immunofluorescence study confirmed that si-Elk1 or si-MMP-9 ameliorated the intracellular distribution of E-cadherin and occludin caused by cyclic stretch (Fig. 3M).

### Elk1 was shown to regulate MMP-9 in VILI

**Fig. 4:**
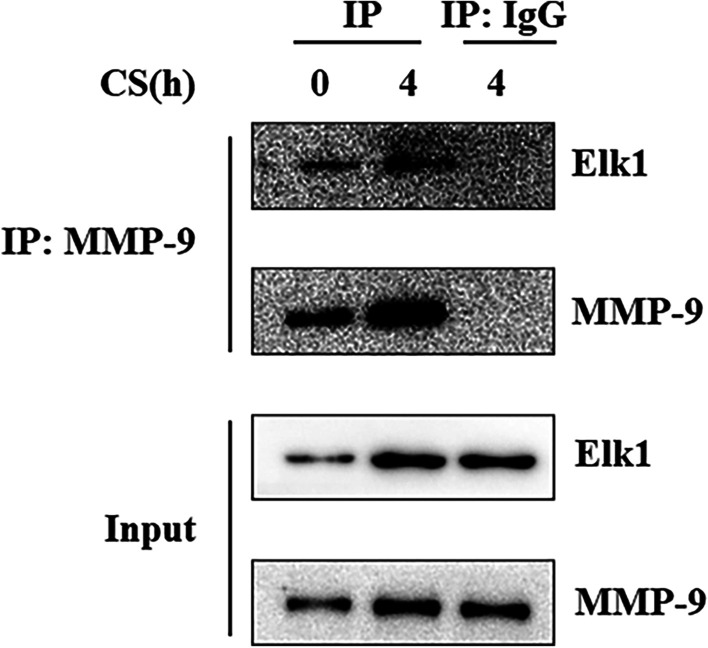
The combination of Elk1 and MMP-9 in MLE-12 cells. The combination of Elk1 and MMP-9 was detected by immunoprecipitation after 20% cyclic stretch for 0, 4 h. Input: Representative Western blotting of Elk1 and MMP-9 in MLE-12 cells treated with cyclic stretch 0 and 4 h. IP: Representative Immunoprecipitation of Elk1 and MMP-9 in MLE-12 cells treated with cyclic stretch 0 and 4 h. MMP-9 and Elk1 were detected from MMP-9 immunoprecipitation, and there were same trend. MMP-9 and Elk1 were not detected from IgG immunoprecipitation

**Fig. 5:**
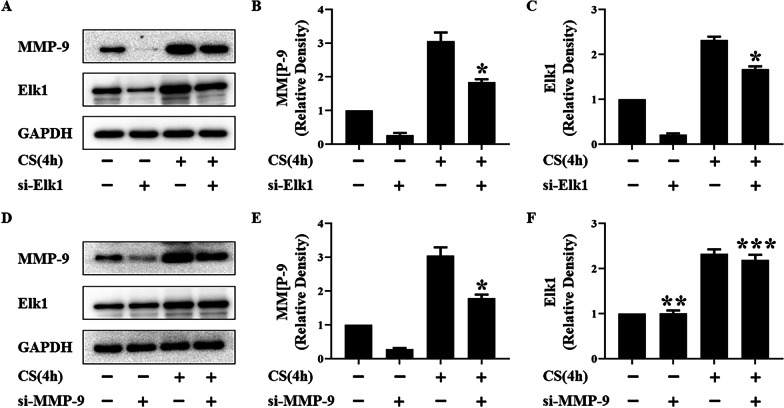
Expressions of Elk1 and MMP-9 in MLE-12 cells treated with or without si-Elk1 or si-MMP-9 or 20% cyclic stretch. **A** and **D** Representative Western blotting of Elk1 and MMP-9 in MLE-12 cells. **B**–**F** The density of the proteins at the control group was used as a standard. *P < 0.05, vs. the CS(4 h) group. **P > 0.05, vs. the control group. ***P > 0.05, vs. the CS(4 h) group

Immunoprecipitation was used to detect the physical interaction of Elk1 and MMP-9 (Fig. 4). Si-Elk1 or si-MMP-9 were used to investigate the relationship between Elk1 and MMP-9 in vitro. The expressions of Elk1 and MMP-9 were measured via western blotting. The expression of MMP-9 decreased when MLE-12 cells were treated with si-Elk1 and subjected to cyclic stretch, when compared to normal cells. The expression of Elk1 was not affected by MMP-9 downregulation (Fig. 5).

### Role of Elk1 and MMP-9 in regulating VILI in vivo

**Fig. 6:**
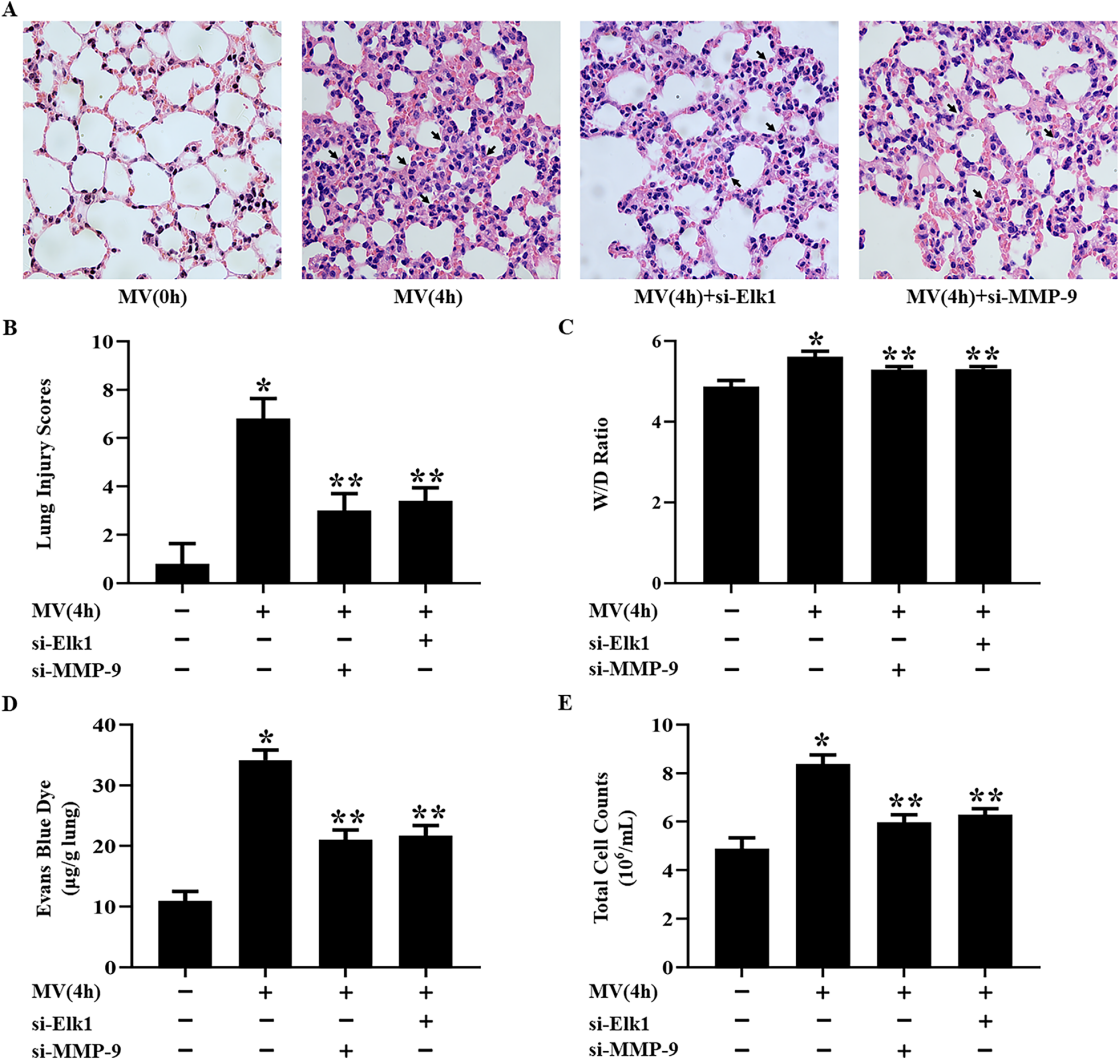
si-Elk1 or MMP-9 ameliorated the pulmonary edema and lung injury in VILI. **A** The pathological changes of lung tissues were determined by HE staining (magnification 400 ×). **B** lung injury scores, **C** W/D weight ratio, **D** Evans blue dye, **E** Total cell counts were used to evaluate the degree of pulmonary edema and lung injury in different group. *P < 0.05, vs. the MV(0 h) group. **P < 0.05, vs. the MV(4 h) group. All experiments were repeated at five times

Mice were treated with si-Elk1 or si-MMP-9 and subjected to mechanical ventilation 4 h. HE staining of lungs showed that edema, atelectasis, necrosis, inflammation, hemorrhage, and hyaline membrane formation were aggravated by mechanical ventilation, compared to healthy mice. These phenomena were reversed by si-Elk1 or si-MMP-9. Total cell counts, lung injury scores, W/D weight ratio, and Evans blue dye extravasation showed that high tidal mechanical ventilation could cause lung injury, while si-Elk1 or si-MMP-9 ameliorated the lung injury (Fig. 6).

**Fig. 7:**
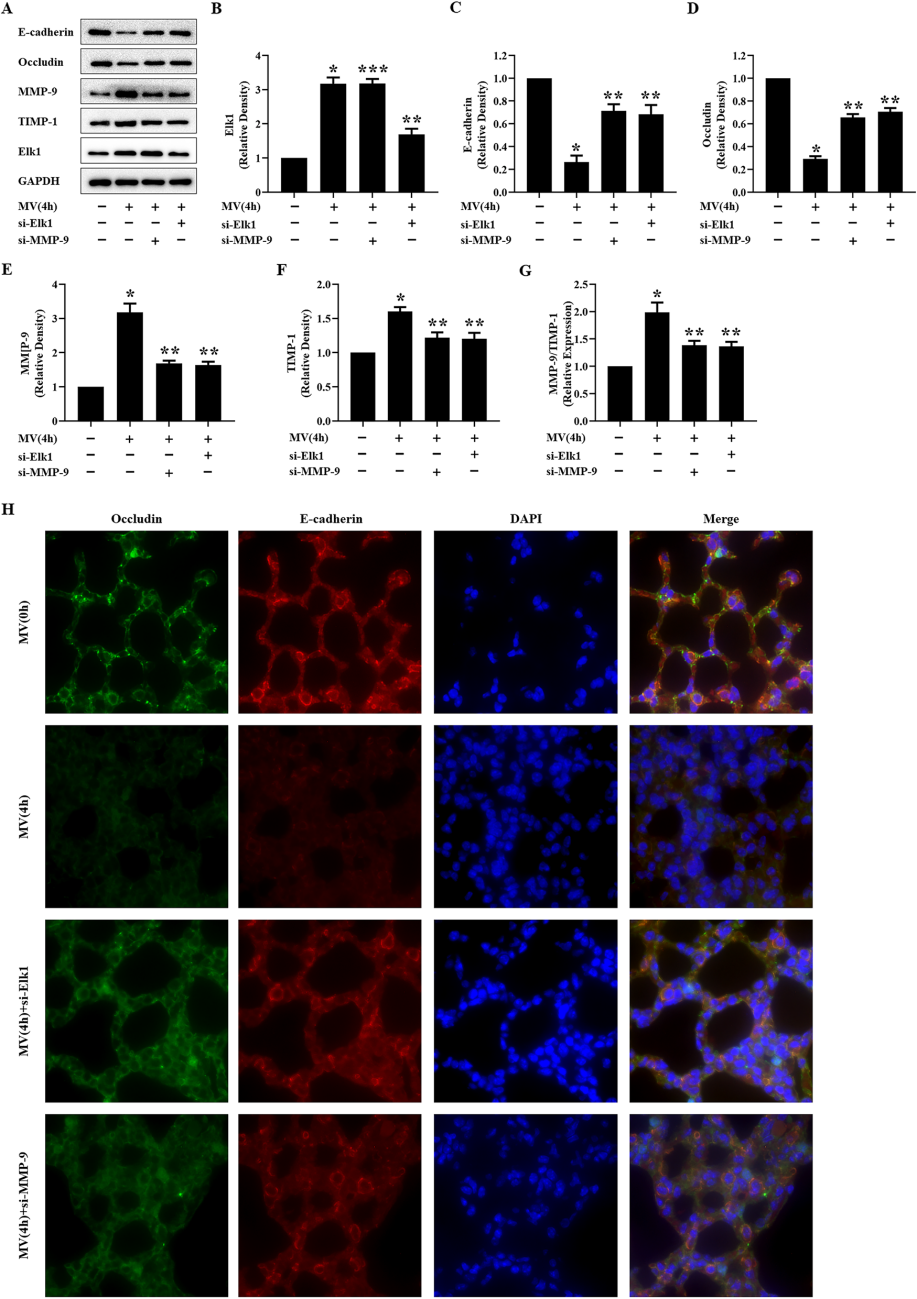
**s**i-Elk1 or MMP-9 ameliorated the expressions of E-cadherin, occludin, Elk1, and MMP-9. **A** Representative Western blotting of E-cadherin, occludin, Elk1, MMP-9, and TIMP-1 in MLE-12 cells. **B**–**F** The density of the proteins at the control group was used as a standard. **G** Ratio of MMP-9/TIMP-1. *P < 0.05, vs. the control group. **P < 0.05, vs. the MV(4 h) group. ***P > 0.05, vs. the MV(4 h) group. H: Immunofluorescence was used to detected the distribution of occludin (green) and E-cadherin (red), and the nuclei were stained with DAPI (blue) in C57BL/6 treated with si-Elk1 or si-MMP-9 and high tidal volume for 4 h. All experiments were repeated at five times

The expressions of E-cadherin, occludin, Elk1, MMP-9, TIMP-1 were measured by western blotting. The expressions of MMP-9 and TIMP-1 and the ratio of MMP-9/TIMP-1 increased in mice treated with high tidal volume mechanical ventilation for 4 h. The expressions of E-cadherin and occludin increased, the expressions of MMP-9 and TIMP-1 and the ratio of MMP-9/TIMP-1 decreased in mice treated with si-Elk1 or si-MMP-9 subjected to high tidal volume mechanical ventilation. Both Elk1 and MMP-9 were activated in mice subjected to high tidal volume mechanical ventilation. Meanwhile, the expression of MMP-9 decreased when mice were treated with si-Elk1. In contrast, the levels of Elk1 were similar in mice treated with or without si-MMP-9. Immunofluorescence analysis evidenced that intracellular E-cadherin and occludin were observed in mice treated with si-Elk1 or si-MMP-9 and high tidal volume mechanical ventilation (Fig. 7).

## Discussion

The current study demonstrates the roles of Elk1 and MMP-9 in regulating tight junctions on VILI models in vivo and in vitro. The major findings of this study are: (1) High tidal volume and 20% cyclic stretch caused VILI in vivo and in vitro, respectively; (2) Elk1 and MMP-9 contributed to tight junctions on VILI both in vivo and in vitro; (3) downregulation of Elk1 or MMP-9 alleviated VILI and (4) Elk1 could regulate the ratio of MMP-9/TIMP-1.

Mechanical ventilation can cause ARDS if not used properly in anesthesia and intensive care units [24]. Thus, it is important to study the underlying mechanisms of VILI. The VILI model in mice and MLE-12 cells previously reported by our team was used to simulate ventilator-induced lung injury seen in the clinic [25]. This study demonstrated that high tidal volume mechanical ventilation caused lung injury as assessed by lung injury scores, W/D weight ratio, Evans blue dye extravasation, and HE staining. The mechanisms herein assessed include activation of inflammatory responses; barrier function damage and changes in sodium, potassium, and calcium plasma channels. This study sought to investigate the mechanisms regulating barrier function.

The alveolar epithelial barrier plays an important role in lung barrier function since it maintains lung tissue homeostasis, participates in pathogen defense, and regulates immune responses [26, 27]. Tight junctions are an integral part of the lung barrier. occludin and E-cadherin are typical proteins of tight junctions. Thus, we used occludin and E-cadherin as indicatives of the alveolar epithelial barrier. In this study, we showed that high tidal volume mechanical ventilation and 20% cyclic stretch could decrease the expressions of E-cadherin and occludin, thus damaging the alveolar epithelial barrier function in a time-dependent manner. These findings are consistent with previous studies. However, underlying mechanisms should be further investigated.

The main function of MMP-9 is to damage and reshape the dynamic balance of the extracellular matrix [14]. MMP-9 plays an important role in degradation and remodeling of the alveolar–capillary membrane and maintenance of the integrity of the basement membrane [28]. Several recent studies have found that MMP-9 is involved in the expressions of E-cadherin or occludin in cerebral ischemia–reperfusion injury, acute lung injury, and acute kidney injury [29–31]. Thus, we investigated if MMP-9 could regulate tight junctions in VILI. TIMP-1 is an MMP9-specific tissue inhibitor, which combines with MMP-9 in a 1:1 ratio [17]. The proportion of MMP-9/TIMP-1 may affect synthesis and degradation of E-cadherin or occludin, which maintains barrier function [32]. The high ratio of MMP-9/TIMP-1 destroyed the lung tissue structure [18]. Our study showed that the high ratio of MMP-9/TIMP-1 decreased E-cadherin and occludin in VILI in vivo. MMP-9 knockdown decreased the ratio of MMP-9/TIMP-1 and reversed the expressions of E-cadherin and occludin to alleviate the lung structure in VILI. Our findings indicate that mechanical stretch could activate MMP-9 to break the alveolar barrier integrity, thus leading to the loss of E-cadherin and occludin. The inhibition of MMP-9 could reverse the above phenomena.

Many recent studies showed that Elk1 plays an important role to regulate tight junctions in acute lung injury and intestinal epithelial barrier dysfunction [33, 34]. Our current findings suggest that Elk1 could be activated in VILI since Elk1 knockdown could increase the expressions of E-cadherin and occludin. Both MMP-9 and Elk1 participated in VILI, and the relationship between these two proteins was still unclear. Elk1 enhanced its binding activity with DNA through its ETS domain, while the MMP-9 promoter region has ETS binding sites. We speculated that MMP-9 and Elk1 interact. Our findings showed that mechanical stretch could increase the physical interaction of MMP-9 and Elk1. We used si-MMP-9 and si-Elk1 to confirm that Elk1 levels were not affected in si-MMP-9 cells subjected to cyclic stretch. However, si-Elk1 could knockdown the expression of Elk1 and MMP-9 at the same time. Our study also showed that Elk1 knockdown decreased the high ratio of MMP-9/TIMP-1 caused by mechanical ventilation. These results indicated that mechanical ventilation upregulated Elk1 to increase MMP-9/TIMP-1 ratio in VILI.

## Conclusions

In conclusion, this study clarifies mechanical stretch damages the tight junctions and aggravates the permeability in VILI, Elk1 plays an important role in affecting the tight junctions and permeability by regulating the balance of MMP-9 and TIMP-1, thus indicating the therapeutic potential of Elk1 to treat VILI.

## Data Availability

Not applicable.

## Abbreviations

VILI: Ventilator-induced lung injury
Elk1: ETS-domain containing protein
MMP-9: Matrix metalloproteinase 9
TIMP-1: Tissue inhibitor of metalloproteinase 1
siRNA: Small interfering RNA
HE: Hematoxylin Eosin
BALF: Bronchoalveolar lavage fluid
DAPI: 4',6-Diamidino-2-phenylindole
BSA: Albumin from bovine serum
RIPA: Radio immunoprecipitation assay
PMSF: Phenylmethanesulfonyl fluoride
ANOVA: One-way analysis of variance
